# Anti-tumor Necrosis Factor-Alpha (TNF-α)-Induced Hypertrophic Lichen Planus: A Case Report

**DOI:** 10.7759/cureus.83491

**Published:** 2025-05-05

**Authors:** Ahmed Alqefari, Ghaida B AlQefari, Sarah Alkhezzi, Sarah Mohammed

**Affiliations:** 1 Medicine and Surgery, Qassim University, Buraydah, SAU; 2 Medicine, Qassim University, Buraydah, SAU; 3 Dermatology, King Fahad Medical City, Riyadh, SAU; 4 Anatomic Pathology, King Fahad Specialist Hospital, Cairo, EGY

**Keywords:** adalimumab, anti-tnf-α therapy, autoimmune disease, case report, dermatologic adverse event, drug-induced lichen planus, hypertrophic lichen planus, immunomodulatory therapy, lichen planus, tnf-alpha inhibitors

## Abstract

We report the case of a 26-year-old male patient with a six-year history of inflammatory bowel disease who developed pruritic, hyperpigmented, and hyperkeratotic nodules on his left abdomen three months after switching from adalimumab to infliximab therapy. Clinical examination and histopathological analysis confirmed the diagnosis of hypertrophic lichen planus (HLP), a rare variant of lichen planus characterized by thick, scaly plaques and intense pruritus. Notably, this adverse reaction represents only the second documented instance of anti-tumor necrosis factor-alpha (TNF-α)-induced HLP, highlighting the potential paradoxical cutaneous reactions associated with these therapies. Although the patient’s inflammatory bowel disease was well controlled with infliximab, the skin lesions responded only minimally to topical corticosteroids. This case underscores the importance of vigilant monitoring of unusual dermatologic manifestations in patients undergoing anti-TNF-α treatment, particularly because such adverse effects can lead to significant morbidity and may be associated with an increased risk of cutaneous squamous cell carcinoma. The mechanisms underlying this paradoxical reaction remain unclear but may involve a shift in cytokine balance following TNF-α inhibition. Increased clinician awareness and early detection are crucial for managing rare drug-induced eruptions and optimizing therapeutic outcomes.

## Introduction

Lichen planus (LP) is a chronic inflammatory condition that primarily affects the skin, mucous membranes, nails, and hair. Clinically, it is characterized by distinctive polygonal, violaceous papules that are often intensely pruritic [1]. Hypertrophic LP (HLP), also known as LP verrucosus, is a rare variant of LP that presents as thick, hyperkeratotic plaques and scales. These lesions are typically extremely pruritic and most commonly appear symmetrically on the shins or the dorsal aspects of the feet. HLP lesions are often persistent, with an average clearance time of approximately six years [1,2]. Tumor necrosis factor-alpha (TNF-α) inhibitors are biologic agents used to treat immune conditions, such as inflammatory bowel disease (IBD), psoriasis, and rheumatoid arthritis, especially when they do not respond to traditional treatments [2]. Many cutaneous and mucosal lesions associated with anti-TNF-α therapy have been described, some of which are paradoxical side effects. TNF-α inhibitors have been successfully used for the treatment of LP [3]. It has recently become apparent that the paradoxical side effect of such biological therapies is a lichenoid reaction [4]. To date, HLP induced by TNF-α inhibitors has been rarely reported in the literature. Only one previous case of adalimumab-induced HLP has been documented [5]. In this report, we describe a second case of HLP triggered by anti-TNF-α therapy, highlighting the importance of recognizing this rare but clinically significant adverse reaction in dermatologic and gastroenterologic practice.

## Case presentation

**Figure 1:**
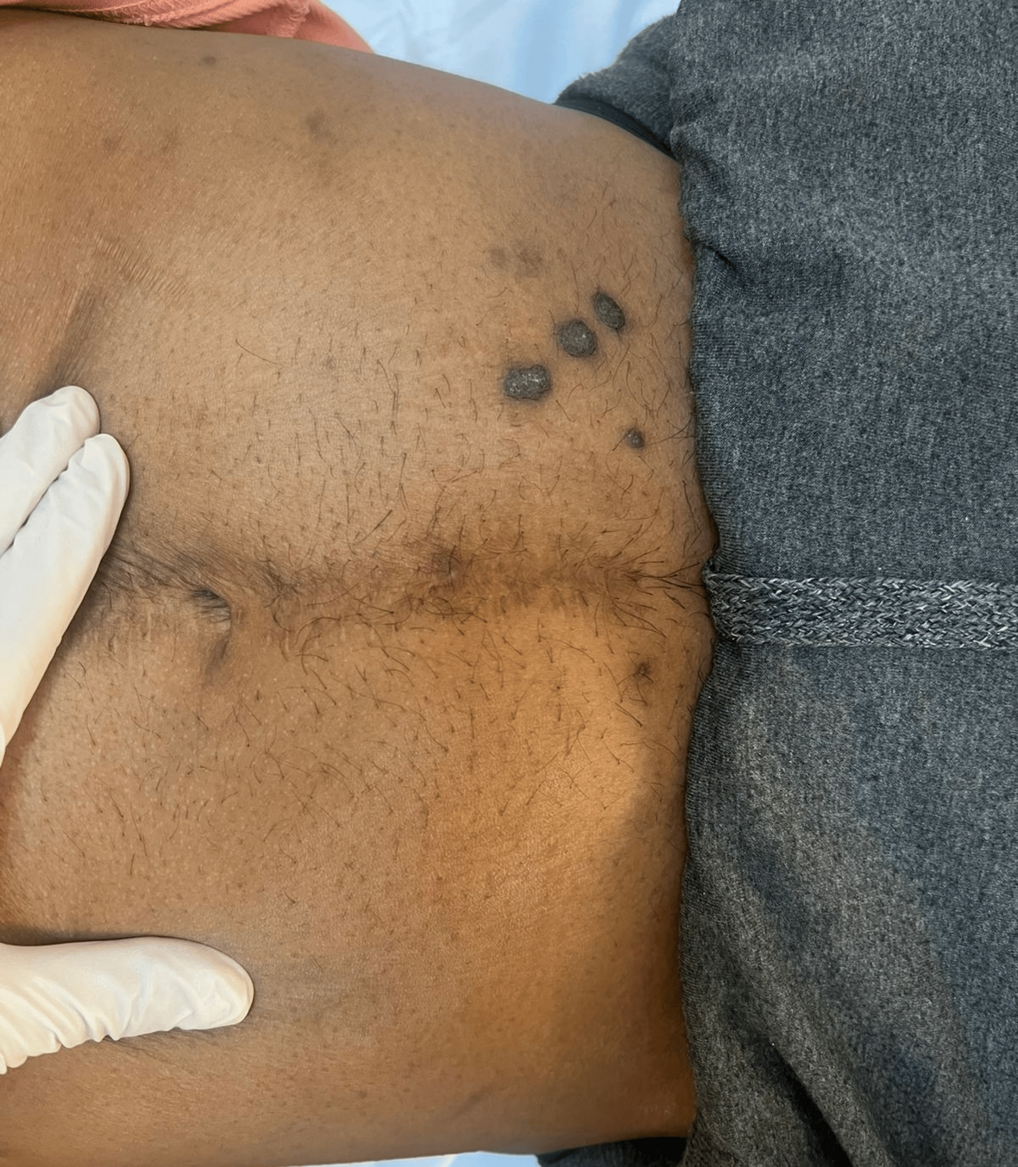
Multiple well-demarcated, polygonal, hyperpigmented, verrucous papules with scaly surfaces, localized on the left lower quadrant of the abdomen.

**Figure 2:**
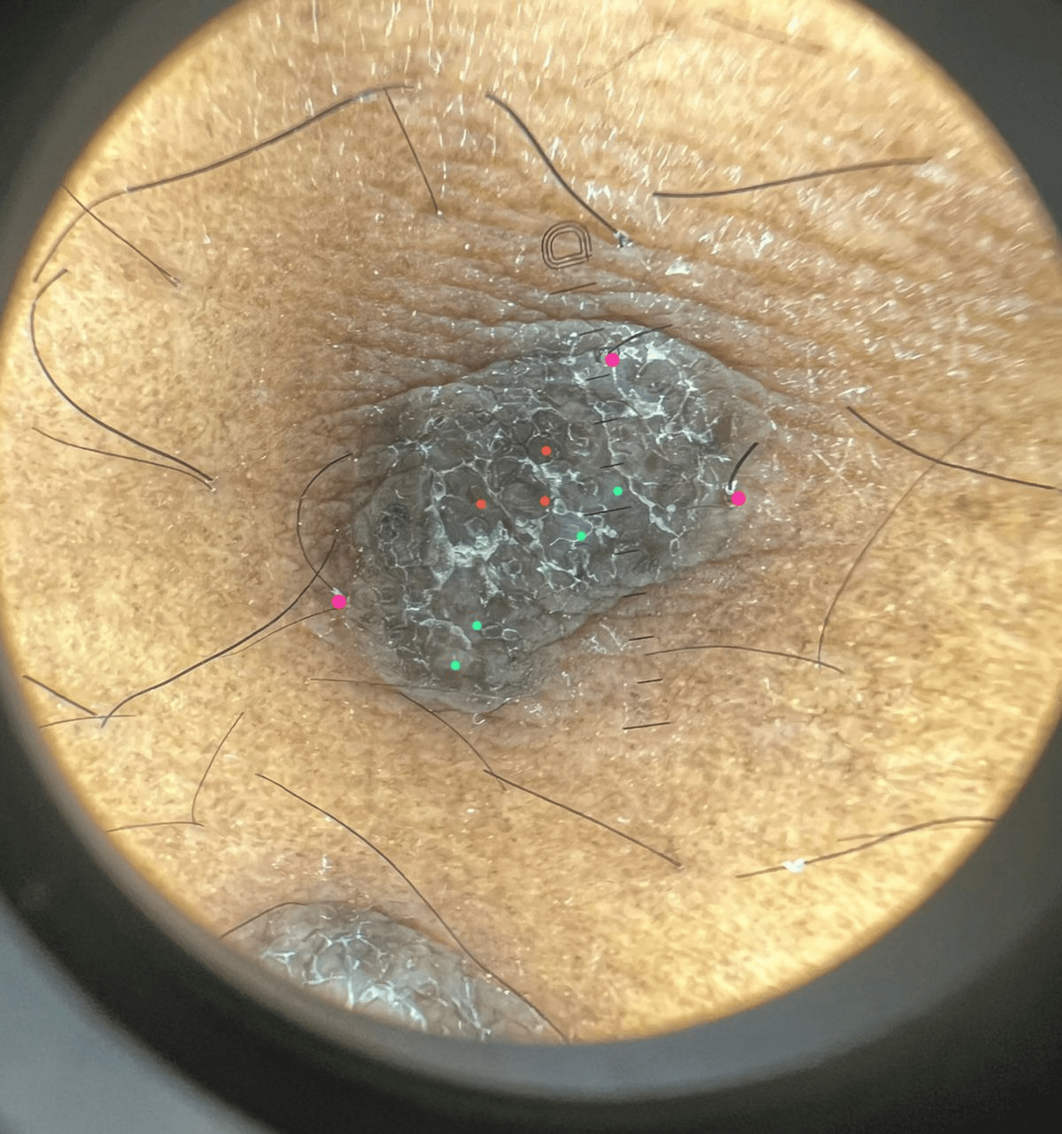
Dermoscopy of the abdominal lesions revealing white areas with minimal striations (green), gray-blue globules (red), and prominent perifollicular casts (pink).

**Figure 3:**
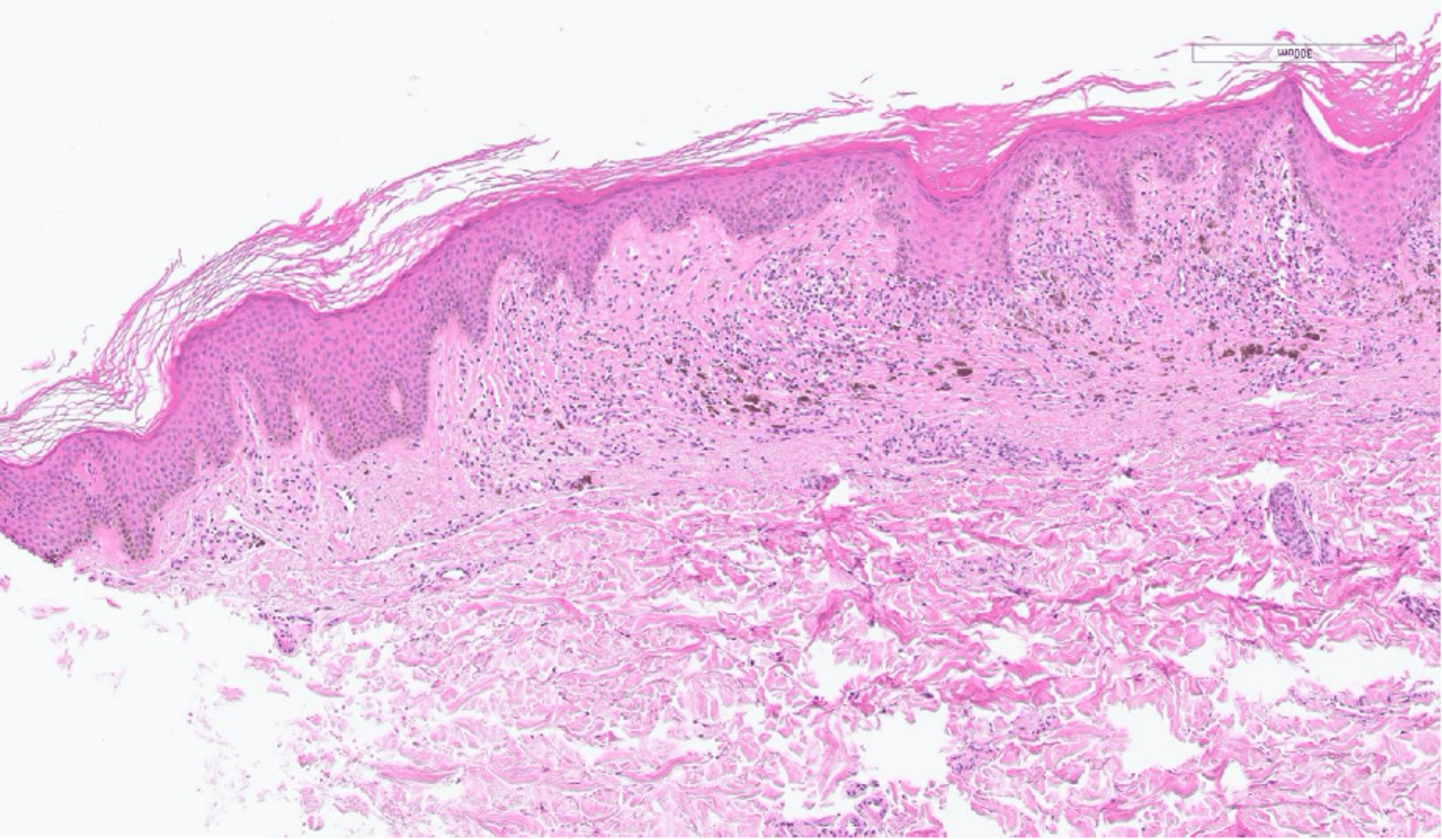
Histopathological section demonstrating features consistent with lichenoid tissue reaction, including mild epidermal thickening, irregular acanthosis, a lichenoid lymphocytic infiltrate tagging the rete ridges, and pigment incontinence (H&E stain). Diagnosis of hypertrophic lichen planus was made through clinicopathological correlation.

**Figure 4:**
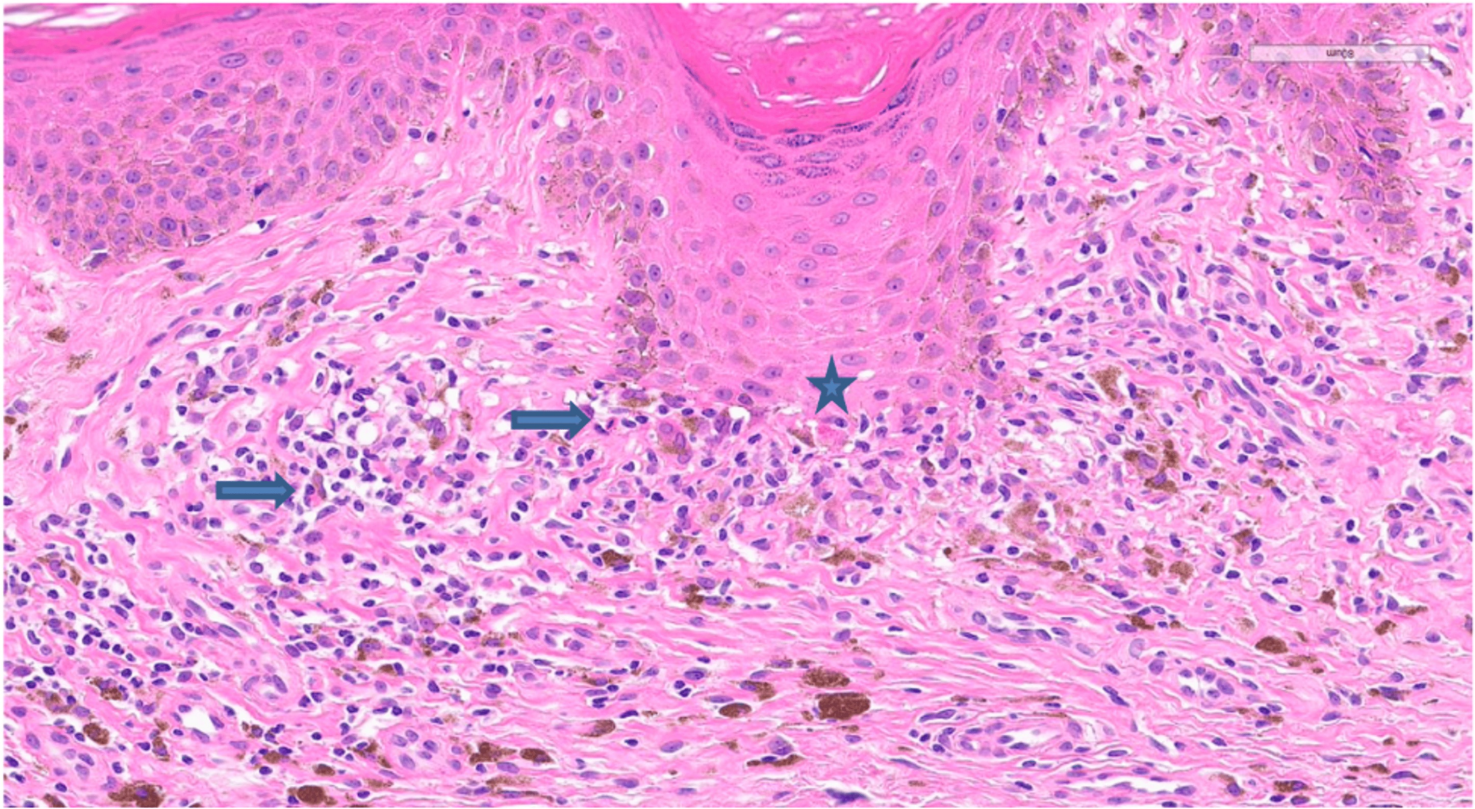
Higher magnification histological view showing follicular epithelium of infundibulum with retained granular layer and keratin plug, a concentrated interface reaction at the rete ridges, colloid body (indicated by a star), and scattered eosinophils (arrows) (H&E stain).

A 26-year-old male patient with a six-year history of IBD, previously managed by adalimumab, presented with multiple hyperpigmented hyperkeratotic nodules in the left abdominal quadrant accompanied by pruritus for the past three months (Figures 1, 2). Due to suboptimal disease control, therapy was later switched to infliximab. At the time of presentation, he had received four doses of infliximab. There was no involvement of the mucosal surfaces, hair, or nails. Histopathological examination of the lesion showed a lichenoid tissue reaction, which is not contradictory with HLP (Figures 3, 4). Based on the correlation of clinical history, physical examination, and histological findings, a diagnosis of TNF-α inhibitor-induced HLP was established. Despite good IBD control with infliximab, HLP showed only minimal improvement with topical corticosteroid therapy. It remains unclear whether this adverse reaction is induced by adalimumab, infliximab, or both, as these agents belong to the same drug class. 

## Discussion

TNF is an inflammatory cytokine produced by a wide variety of cell types, including keratinocytes and macrophages. It plays a central role in innate immunity and host defense. The immunological mechanisms underlying HLP involve dysregulation in cytokine production, notably TNF-α, which can activate T cells and dendritic cells, leading to lichenoid eruption [1,6]. Moreover, interference with the interleukin (IL)-4/IL-13 signaling pathway-such as through targeting the IL-4 receptor alpha chain-can downregulate T-helper 2 (Th2) activation, shifting the immune balance toward Th1 dominance. Lichenoid drug eruptions are considered Th1-mediated dermatoses [7].

The paradoxical exacerbation of LP symptoms upon the administration of TNF-α inhibitors (adalimumab, infliximab, golimumab, and certolizumab) reinforces the complex interplay between TNF-α and LP, suggesting that while TNF-α inhibitors are typically effective in treating inflammatory conditions, they may inadvertently trigger LP lesions in susceptible individuals [8]. Research indicates that individuals with oral LP (OLP) demonstrate elevated levels of TNF-α, correlating with the severity of the lesions [9]. The systemic release of cytokines, including TNF-α, may contribute to the disease's clinical presentation through effects on keratinocyte proliferation and immune regulation [10]. In the context of TNF-α inhibition, the unexpected development of HLP raises intriguing therapeutic dilemmas, as clinicians may encounter patients who respond paradoxically with cutaneous lesions to drugs meant to modulate their autoimmune conditions [11]. Interestingly, interferon-alpha upregulation has been proposed as a key mechanism behind paradoxical inflammatory reactions, such as the induction of psoriasis in patients treated with TNF-α inhibitors. It is possible that a similar immunological pathway contributed to the development of HLP in this patient [12].

Our patient developed HLP shortly after transitioning from adalimumab to infliximab, both of which are TNF-α inhibitors. Adalimumab is a fully human monoclonal antibody, whereas infliximab is a chimeric antibody. While the exact triggering agent remains uncertain, the shared drug class and timing support a strong causal association. An eight-week interval between the third and fourth doses of infliximab may have led to a decrease in blood levels of the drug, which could have contributed to the development of paradoxical LP. This possibility adds complexity to the understanding of this reaction, although other immune-related factors may also have played a role.

To date, only one previous case of adalimumab-induced HLP has been reported, involving a patient with psoriatic arthritis who developed lesions on the hands and forearms [5]. Our report represents the second known case of anti-TNF-α-induced HLP. Since 2002, several cases of TNF-α inhibitor-induced lichenoid eruptions-including cutaneous LP, OLP, and lichen planopilaris-have been documented in the literature [11-14]. These paradoxical reactions may cause significant patient discomfort and also carry a risk of malignant transformation, particularly in long-standing HLP lesions [15].

Given the expanding use of TNF-α inhibitors in clinical practice, dermatologists and other clinicians should remain vigilant for such rare adverse effects. Early identification and histopathologic confirmation are essential for timely management and for minimizing potential complications. Regular skin examinations should be considered for patients receiving long-term biologic therapies, especially when new or persistent skin lesions emerge.

## Conclusions

This case highlights a rare but clinically significant paradoxical reaction to TNF-α inhibitors in the form of HLP. Although these biologics are widely used to manage immune-mediated conditions such as IBD, they can occasionally trigger unexpected cutaneous adverse effects. Recognizing such manifestations is crucial, as delayed diagnosis may lead to prolonged morbidity and an increased risk of complications, including malignant transformation. Clinicians should maintain a high index of suspicion for drug-induced eruptions in patients receiving TNF-α inhibitors and consider histopathologic confirmation when lesions persist or display atypical features. Early identification and appropriate management are essential for optimizing patient outcomes.
